# Social drivers of sleep experiences: Conversations with midlife working-class women from Mexico city

**DOI:** 10.1017/slp.2025.5

**Published:** 2025-04-24

**Authors:** Astrid N. Zamora, Elizabeth F.S. Roberts, Martha M. Tellez-Rojo, Karen E. Peterson, Libni A. Torres-Olascoaga, Alejandra Cantoral, Erica C. Jansen

**Affiliations:** 1 Department of Epidemiology and Population Health, Stanford University School of Medicine, Stanford, CA, USA; 2 Department of Nutritional Sciences, University of Michigan School of Public Health, Ann Arbor, MI, USA; 3 Department of Anthropology, University of Michigan, Ann Arbor, MI, USA; 4 Center for Research on Nutrition and Health, National Institute of Public Health, Cuernavaca, Mexico; 5 Health Department, Ibero-American University, Mexico City, Mexico; 6 Department of Neurology, Division of Sleep Medicine, Michigan Medicine, Ann Arbor, MI, USA

**Keywords:** ethnography, epidemiology, global health, midlife, mixed-methods, sleep

## Abstract

Sleep is essential for the health of midlife women, yet the barriers (factors that impede) and facilitators (factors that support) to achieving adequate sleep, particularly among working-class women in Mexico City and broader Latin American contexts, remains insufficiently understood. This study aims to provide a nuanced understanding of the factors influencing sleep among working-class midlife women in Mexico City. A mixed-methods approach, combining quantitative data (epidemiologic measures) and qualitative data (ethnographic interviews), was employed among women enrolled in a Mexico City cohort. We used epidemiologic data to describe sleep and its correlates in a sample of 120 women, incorporating both self-reported (questionnaires and sleep diaries) and behavioral (actigraphy-based) measures of sleep. A subset of 30 women participated in in-depth ethnographic interviews to explore determinants of sleep, including barriers, facilitators and coping strategies to compensate for sleep loss. Our findings reveal that many women experienced poor sleep, with 43% reporting insomnia-related difficulties and 53% experiencing short sleep duration. Barriers included family-related stress, particularly caregiving responsibilities, economic instability, and mental health challenges. In response to sleep loss, women often resorted to coping mechanisms, such as caffeine consumption and napping, and the use of natural remedies. This study highlights the critical role social factors, including family dynamics and caregiving roles, in shaping sleep health outcomes. Sleep, as an inherently social behavior, is strongly influenced by these contextual factors. These findings underscore the importance of considering psychosocial and cultural contexts in interventions aimed at promoting healthy sleep in midlife women.

## Introduction

Sleep impacts nearly all aspects of health and well-being. Public health recommendations provide guidelines on the duration of sleep needed to sustain health as well as hygiene principles that encourage adequate sleep quality (Consensus Conference Panel et al. 2015). Sleep hygiene principles include maintaining consistent bedtimes and wake times, sleeping in a cool, dark and quiet space and avoiding screens before bed (Irish et al. 2015). These guidelines often assume that individuals have full control over their sleep environment and habits. Yet, this perspective overlooks broader psychosocial and cultural context factors that can significantly influence sleep outcomes. While these sleep recommendations provide seemingly straightforward guidance at the population level, they may only resonate with specific demographics across certain life stages. For example, parents of newborns are unlikely to achieve adequate sleep duration or to be able to enact all sleep hygiene principles (Richter et al. 2019); thus, sleep interventions must be appropriately tailored and modified to meet the needs of specific populations and take into account particular barriers to sleep.

Anthropology, among other fields, emphasizes the need to situate health behaviors and public health recommendations within specific populations’ lived experiences and social realities. Ethnography, involving long-term participant observation and in-depth interviews, provides a broad aperture for discerning what matters within a particular social ecology (Goodson and Vassar 2011). For example, instead of asking directly about the specific effects of home environment exposures (e.g., light or noise) on sleep, an ethnographer might observe how a group, in general, describes sleep, what sleep does and what typical sleep looks like in order to paint a picture of sleep within the population. More recently, combining ethnographic and epidemiologic methods has provided insights into complex psychosocial phenomena, including sleep behaviors (Roberts 2021).

Mixed-methods research approaches are needed to develop a better understanding of sleep and its determinants within a particular ecology, especially among populations experiencing sleep disparities. Midlife women may be especially at risk of sleep disparities, including short sleep duration, poor sleep quality and sleep disorders such as insomnia (Baker et al. 2018; Benge et al. 2024; Smith et al. 2018; Wong et al. 2023). While the menopausal transition has been widely considered a pivotal driver of poor sleep among middle-aged women (Baker et al. 2018) between the ages of 40 and 65 (Thomas et al. 2018), some studies, primarily epidemiologic, have provided conflicting evidence on the role of menopause in sleep during midlife. For instance, a recent longitudinal study indicated that sleep disturbances were a significant issue among midlife women regardless of age or reproductive stage (Jones et al. 2018). Similarly, a separate longitudinal study among participants from the Study of Women’s Health Across the Nation (SWAN) found that multiple markers of sleep health did not worsen over time for midlife women (Matthews et al. 2020). Furthermore, some studies suggest that the menopausal transition itself may not be the primary driver of sleep disturbances during this period of life but rather that other contextual factors present during this stage of life (e.g., job/occupation, financial and psychosocial and cultural context) may be more strongly related to sleep (Dao-Tran and Seib 2018; Luna et al. 2014; Maeda et al. 2020; Shaver and Woods 2015; Sidani et al. 2019).

Within the existing literature on sleep among midlife women, studies conducted among Mexican women are largely absent, with most studies focusing on non-Hispanic white (NHW) women from the United States (US) and European countries or immigrant Latina women residing in the US (Arakane et al. 2011; Gaston et al. 2019; Wu et al. 2020). However, women in Mexico, especially those from urban working-class settings, experience sleep-related challenges that differ from those faced by women in other racial, ethnic, or socioeconomic groups. In particular, extended family households are typical in Mexico, where multiple generations often share sleeping spaces, creating different dynamics around sleep compared to women in Western contexts where more private sleeping arrangements are typical. Moreover, although it is well-documented that women of the midlife age range often have caregiving roles, this role and its effect on sleep may be even stronger in these multi-generational settings. The other important context to consider in this working-class population, though not necessarily specific to Mexico, is the nature of work, which can include long work hours and non-regular schedules. It is also common for women to be self-employed or to be working for pay but not formally employed. Altogether, these factors suggest that the experiences of sleep among working-class Mexican women may be shaped by specific social, familial and economic dynamics that differ from other populations typically studied in sleep research.

Thus, the aim of this mixed-methods study was to combine ethnographic and epidemiological approaches to provide a nuanced understanding of the ecological factors influencing sleep health in this population, with a particular emphasis on the psychosocial and cultural factors, such as family dynamics and caregiving responsibilities, that may affect sleep. The aims of the study were two-fold: (1) to utilize existing epidemiologic data sources to describe sleep and its correlates in this study population (n=120) via behavioral (actigraphy) measures and self-reported (questionnaires and sleep diaries) sleep measures and (2) to use data from in-depth ethnographic interviews (n=30) to characterize self-described barriers and facilitators of sleep, including strategies women implement to cope with sleep loss. The results of these aims were then used to reflect on the question posed by Sephton and Kay: “How do psychosocial and cultural factors influence sleep and circadian health disparities?” Their work emphasizes the need to explore the psychosocial and cultural determinants that contribute to sleep disparities across different populations (Sephton and Kay 2024). This question is particularly relevant to our study as it aligns with our focus on the experiences of working-class midlife women in Mexico City – a population underrepresented in the literature. By documenting the sleep patterns and associated factors affecting this group, we aim to contribute to a more globally inclusive understanding of sleep health and disparities in midlife women. In doing so, we highlight the importance of considering the psychosocial and cultural context in which sleep occurs, particularly in non-Western, low-income settings where structural and every day realities may uniquely shape sleep experiences.

## Methods

### Sample and recruitment

Women in the present study were recruited into the Early Life Exposure in Mexico to Environmental Toxicants (ELEMENT) birth cohort in 1994 during pregnancy or at delivery from the Mexican Institute of Social Security family clinics, serving a low- to middle-income population in Mexico City, MX. A follow-up study was conducted between 2019 and 2020 (pre-COVID-19 pandemic). At this visit, 120 women (quantitative sample) provided data on demographic characteristics, anthropometry, sleep assessments based on actigraphy and sleep diaries and a general study questionnaire including health history.

Inclusion criteria for the ethnographic study entailed having participated in the 2019–2020 study visit, with women who did not participate in the study visit not eligible for the ethnographic study. To gather data about women’s experiences during pre-, peri- and post-menopause, this study implemented a purposive sampling strategy consistent with achieving a range of ages from 30 to 64 years. Recruitment was conducted over the phone in Spanish by a member of the ELEMENT fieldwork team. The final qualitative (ethnographic) sample comprised a subset of 30 interviewees from the quantitative sample. Recruitment and interviews occurred between June and September 2021. A retention rate of 100% was achieved for all participants who were initially approached to participate in the study.

### Epidemiological measures

Participant demographics were obtained using a self-reported questionnaire administered between 2019 and 2020. Participant demographic characteristics included age, employment status, occupation details, number of bedrooms in the household (a relevant measure included due to a previous ethnographic study conducted among the population), parity, household socioeconomic status (SES), household food insecurity (HFI) status and smoking behavior. We also obtained information on health history. Finally, we estimated sedentary time from wrist-actigraphy devices.

Age (years) was categorized into the following tertiles: 33–42 years, 43–48 years and 49–60 years. Employment status was categorized as employed (formally or informally), unemployed, retired/pensioned, or dedicated to the household full-time. Participants self-reported the number of bedrooms in the household, and we further operationalized the variable into the following categories: 1, 2, 3 and ≥ 4 bedrooms. Parity (a proxy for the number of children living in the household) was operationalized as ≤ 1, 2 and ≥ 3. Household socioeconomic status (SES) was self-reported and assessed using a 10-item region-specific household-based survey that was developed and index standardized (i.e., AMAI 8 × 7) by the Mexican Association of Marketing Research and Public Opinion Agencies (AMAI) to classify the SES of the Mexican population (López Romo 2009). These covariates and self-reported HFI were obtained from a standard questionnaire, and the classification of smoking behavior has been previously described elsewhere (Zamora et al. 2021).

For self-reported health measures, we focused on diabetes, mental health and menopausal status. We assessed the presence of diabetes using the following close-ended, yes/no questions: (1) “Have you been told by a doctor that you have diabetes or high blood sugar?” and (2) “Has a doctor told you that you have prediabetes or higher than normal blood sugar levels?”. Next, we operationalized the variables into three categories: yes, no (for responses obtained from question 1), or pre-diabetic (for “yes” responses obtained from question 2). The presence of a mental health condition (yes or no) was dichotomized based on responses to the following close-ended, yes/no question: “Have you ever been diagnosed by your doctor or treated for a mental/psychiatric condition?”. Validated methods and procedures were used to determine the menopausal status (Johnston et al. 2006) based on an algorithm incorporating age at the time of hormone collection, last reported menstrual cycle and one-time measures of Follicle-Stimulating Hormone (FSH) and Estradiol (E2) hormone levels (Johnson et al. 2004). We obtained four categories for menopausal status: peri-menopausal, pre-menopausal, post-menopausal, or surgical menopause. Finally, sedentary time was derived via actigraphy and measured in hours per day based on response to the open-ended question: “During the last seven days, how much time did you spend sitting on one day in the week?”.

### Self-reported (questionnaires and sleep diaries) and behavioral (actigraphy-based) measures of sleep

All sleep measures are described in Figure 1. Briefly, self-reported variables were obtained from two sources: (1) a general sleep questionnaire obtained at the 2019–2020 study visit and (2) sleep diaries completed by participants over seven consecutive days following the 2019–2020 study visit, coincident with when they wore the actigraphy. In addition, behavioral measures of sleep were estimated using wrist-actigraphy devices (ActiGraph GT3Xþ; ActiGraph LLC, Pensacola, FL) that participants wore on the non-dominant wrist for seven consecutive days. Trained personnel placed an actigraphy device on the participant’s wrist at the end of the study visit. Weekly nightly sleep measures were estimated from actigraphy data using a pruned dynamic programming (PDP) algorithm developed by R (R Foundation for Statistical Computing, Vienna, Austria). The PDP approach incorporates self-reported bedtimes and wake times to improve accuracy (Baek et al. 2021). A description of the variables measured with the device is listed in Figure 1.

### Ethnographic qualitative data collection

We conducted ethnographic interviews as part of our data collection process. Two female ELEMENT researchers, both trained in ethnographic fieldwork methods by an anthropologist (EFSR, Doctor of Philosophy in Anthropology), conducted the interviews in Spanish. At the time of the interviews, the lead interviewer (ANZ, Master of Public Health) was a doctoral candidate at an accredited school of public health within the US, while the second interviewer, who also identified as female, had extensive experience working in the field with ELEMENT participants prior to the start of this ethnographic study. This prior involvement with the community allowed the interviewers to establish rapport and trust, which is a key component of ethnographic research (Hammersley and Atkinson 2019). The interviews were conducted in an ethnographic style, focusing on understanding participants’ lived experiences within the psychosocial and cultural context of their daily lives, including their social, familial and environmental environments. Although we had a set of core topics to explore, the interview process was semi-structured and flexible, allowing participants to guide the discussion. Rather than adhering to strict question order, the interviews prioritized topics that participants felt were most important, with the freedom to explore those themes for as long as they wished. This approach reflects the ethnographic emphasis on context and participant-driven inquiry (Spradley 1979). The goal of this method is not only to capture participants’ verbal responses but also to gain insight into the emotional and contextual layers of their experiences through active, reflective engagement between the interviewer and participant. Every one-time interview lasted approximately 45–60 minutes and was conducted via Zoom audioconferencing. During the interviews, we focused on topics such as sleep environment and family dynamics, sleep behaviors and beliefs, sleep history and self-perceived barriers and facilitators of sleep. We also explored the impact of COVID and menopause on sleep.

Immediately after each interview, the researchers wrote detailed fieldnotes documenting their experiences during the interview, including their observations, feelings and reflections on the conversations. This practice is a key element of ethnographic research, where fieldnotes are used to capture the context of the interview and the researcher’s position in the data collection process (Emerson et al. 2011). The fieldnotes allowed us to capture emotional nuances, tone of voice and the broader social dynamics at play during the interviews, which are critical elements of understanding participants’ lived experiences in ethnography. The fieldnotes, along with the verbatim transcriptions of the interviews, were used to analyze the data holistically, considering both the content and context of the participants’ responses.

The interview recordings were transcribed verbatim in Spanish by a native speaker. To protect participants’ anonymity, all names and identifiable information were replaced with pseudonyms following a systematic substitution pattern.

## Statistical analysis

### Stage 1: Quantitative data analysis

We ran descriptive statistics to compare the larger ELEMENT study sample (N=120) to the ethnographic study sample (N=30) across demographic, clinical and sleep characteristics. For each variable, we report the median (IQR) or proportion. We then examined the demographic and clinical correlates of the sleep characteristics in the epidemiologic sample (N=120) using statistical tests to examine associations. These tests included Chi-square tests for categorical variables and Mann-Whitney U tests for continuous variables, as appropriate. We report effect sizes, along with the corresponding p-values to indicate the strength and significance of the findings, with significance set at p < 0.05. All analyses were conducted using SAS 9.4 (Cary, NC, USA).

### Stage 2: Qualitative data analysis

We conducted thematic analysis on interview fieldnotes, which were written in either English or Spanish and exported into ATLAS.ti (Version 9.1.7.0) for coding and analysis. Our research team developed a codebook (Supplemental Table 1) that incorporated both a priori codes informed by our research questions and existing literature and emergent codes identified during data review. Initial independent coding was conducted by one researcher (ANZ), who applied the a priori codes, including key themes such as barriers and facilitators of sleep. Two additional team members (ECJ and EFSR) reviewed these initial codes and contributed to the identifcation and refinement of emergent themes, particularly those related to coping strategies and cultural factors. Coding was reviewed and revised through iterative team meetings, during which discrepencies were discussed and coding decisions were refined by consensus, promoting a collaborative and reflexive approach to theme and subtheme development and minimizing individual coder bias. We then grouped codes into key thematic areas aligned with our research aims, including: (1) perceptions of why sleep is needed, (2) barriers to sleep, (3) facilitators of sleep and (4) strategies women use to compensate for sleep loss. While some themes were developed a priori, one key theme (i.e., strategies to compensate for sleep loss) emerged a posteriori, reflecting the participants’ unique experiences and the unique socio-cultural context in which they were embedded. Emergent subthemes were related to the ways in which sleep is influenced by familial responsibilities, cultural expectations and environmental factors.

Subthemes often overlapped and were not mutually exclusive; for example, familial responsibilities appeared as both a barrier and a motivator for sleep in different contexts. These nuances were captured through flexible coding and inclusive team discussions.

We present both qualitative and quantitative results distinctly. For the quantitative analysis, we highlight statistically significant findings. For the qualitative analysis, we present key themes, subthemes and representative quotes from fieldnotes, along with a summary of the primary findings. Our reporting follows the Consolidated criteria for reporting qualitative research (COREQ) checklist for qualitative research (Tong et al. 2007). In line with the COREQ checklist, we confirm that participants did not provide feedback on the coding process or final results summary.

## Results

Table 1 summarizes the demographic, clinical and sleep characteristics of the epidemiologic sample (N = 120) and the ethnographic sample (N = 30). The epidemiologic sample of 120 women had a mean (SD) age of 46 (8.0) years, with over one-third of the women reporting that they were dedicated to their household full-time, retired, or pensioned at the time of the study. From the actigraphy data, 52.5% had insufficient sleep (duration of < 7 hours per night), 10.8% self-reported experiencing poor sleep quality ≥ 3 days, and 43.3% self-reported experiencing a hard time falling asleep ≥ 3 days. The ethnographic sample had similar characteristics to the overall sample, with a few exceptions, including that the ethnographic sample was slightly older [50.0 (9.0) years], and only 3.3% of the sample reported experiencing poor sleep quality ≥ 3 days during the week of assessment.

Regarding the ethnographic sample, over one-third (37%) reported that they were dedicated full-time to the household or pensioned, 33% reported working in clerical/office work type of occupations, 23% were employed in blue-collar services, and 7% were small business owners. Details about specific job titles/duties are listed in the participant profile table. In addition, based on interviews with women, where they described their jobs/occupations and sleep schedules, we did not find evidence that women in the sample were either shift- or night-shift workers. Women who reported being dedicated full-time to household duties indicated a duration ranging from 2 to 26 years. In addition, 23% of the sample lived in multi-family households, defined as households that included either multi-generational households (e.g., participants living with offspring and their grand offspring or participants living with their parents, etc.) or participants living with multiple families within the same generation (e.g., the participant’s sibling’s family, etc.). Moreover, 23% of the women shared a bedroom with two or more people. Furthermore, we found we found that 47% (14/30) of women mentioned experiencing insomnia in the ethnographic interviews, which aligns with 43.3% of women from the epidemiologic sample who self-reported that they were experiencing a hard time falling asleep for ≥ 3 days during the week of assessment. See Supplemental Table 2 for full participant profiles.

Demographic and clinical correlates associated with self-report and behavioral (actigraphy-based) sleep characteristics among the epidemiologic sample (N=120) are presented in Table 2. Age, type of occupation/work, household food insecurity, presence of diabetes and presence of a mental health condition were related to at least one aspect of sleep health. To illustrate, participants dedicated to their households full-time reported significantly longer sleep duration than their counterparts (p = 0.0044). The type of occupation/work was also significantly correlated with sleep latency, with those dedicated to the household and independent/self-employed business owners having a longer latency of approximately 5 minutes compared to their counterparts (p = 0.0118). Greater sleep variability was associated with having a mental health condition [median (Q1, Q3) = 102.1 (83.8, 115.7) min vs. 73.2 (48.1, 95.1) min; p = 0.002]. In addition, presence of mental health condition(s) was associated with poor sleep quality ≥ 3 days, with 26.7% of those with a mental health condition having poor sleep quality compared to 8.7% of their counterparts who did not have a mental health condition (p = 0.03). Being diabetic or pre-diabetic was correlated with an earlier sleep midpoint compared to those who reported not having diabetes (P = 0.04). A negative linear trend was observed between older age and social jetlag, with participants between 30 and 43 years having a median (Q1, Q3) social jetlag of 1.1 (0.7, 1.7) hours compared to 49–60-year-old women who had a social jetlag of 0.6 (0.2, 1.0) hours (p = 0.01). Actigraphy-based sleep duration and napping ≥ 3 days/week were not associated with demographic or clinical measures. In addition, menopausal status was not associated with any sleep characteristics.

In this study, we explored four major themes related to sleep among women: (1) the perceived importance of sleep, (2) barriers to sleep, (3) facilitators of sleep and (4) strategies women employed to compensate for sleep loss. Within these overarching themes, we identified several subthemes that highlight specific insights shared by participants. Below is a presentation of our findings, beginning with Table 3, which summarizes these subthemes and representative quotes from researcher fieldnotes, providing a deeper understanding of why sleep is needed by the midlife women in this sample.

First, throughout interviews, women discussed sleep as crucial for providing energy and rejuvenating the body. For example, one interviewer’s fieldnotes describe how a participant responded: “It is sleeping that helps you recover so that the body has energy. She also told us that sleep is something that the body asks for. She considers that sleep should be sacred like the food we eat” (Age 49).

The second subtheme that emerged was how sleep prevents poor mental/physical health. For example, in an interview with a 39-year-old participant, fieldnotes captured this idea “She mentioned that if someone lacks sleep, they can experience a heart attack, and their vision and ability to hear can become impaired. She told us she experienced tachycardia from her lack of sleep and thinks sleep is critical for the human race.” The third subtheme was that many women felt that sleep was necessary for work. For example, one interviewer recorded in their fieldnotes: “The last thing she (participant aged 59) said about sleep was that sleep is essential; if you do not sleep enough, you can’t shine at work and people need to sleep to be able to function the next day.” The final subtheme noted that sleep was considered necessary to avoid accidents. For example, one woman vividly described what happened to her at work when she lacked sleep: “When I asked her how her work used to be affected if she lacked sleep, she said that it felt so heavy. Once she burned her hand for being so tired at work and not being able to focus” (Age 56).

Table 4 describes the common subthemes women perceived as sleep barriers faced by themselves or others. Eight barriers were noted, including: (1) family, (2) economic situation, (3) stress/rumination (i.e., repetitive thinking or dwelling on negative feelings and distress), (4) unexplained insomnia, (5) job/occupation schedule or stress, (6) ecological factors (e.g., light, noise, temperature, etc.), (7) medical conditions/illnesses; and (8) caffeine/food. We found that family concerns were the most frequently reported barrier to achieving adequate or high-quality sleep among the sample (barrier 1); examples included obligations related to family members and worrying about family members living within or outside the home. One woman described how worrying about her children impacted her sleep, as reported by an interviewer: “Worrying about her children and their obligations, such as schoolwork that stresses them out or keeps them up at night (is what impacts her sleep)” (Age 47). Fieldnotes from another interview illustrated the following: “Worrying about a family member coming home late from work … which is true for her, since her son gets home late from work … she told us that her daughter gets home super late at night (1:30 AM) and she won’t sleep until her daughter gets home.” (Age 49). Among the sample, economic situation (barrier 2) was the second most described barrier to sleep. From fieldnotes, one interviewer reported, “Some of the factors that impact her sleep include financial concerns, those that affected her immediate household during the pandemic” (Age 51). Another woman mentioned how her financial situation affected her sleep during the day: “She cannot take naps during the day because of her current (financial) situation (Age 60).” In addition, stress/rumination was another significant barrier (barrier 3). This subtheme captured when women described thinking or overthinking as something that created a barrier to sleep, including thoughts about activities for the next day. For instance, a woman described the ruminating thoughts that keep her awake at night. From the interviewer’s fieldnotes: “She repeated to us that she would not sleep because she was really worried about getting sick and stressed” (Age 45).

Unexplained insomnia was the fourth barrier that emerged from interviews with women. This encompassed discussions in which women explicitly mentioned insomnia or symptoms of insomnia, including distress over having a hard time falling or staying asleep without knowing why this occurred. For example, one woman described the following, summarized in fieldnotes: “The participant told us that she used to sleep well at night but has experienced insomnia for the past five years and has difficulty sleeping” (Age 54).

The fifth barrier identified was job/occupation schedule or job-related stress, exemplified by the following excerpt from interviewer fieldnotes: “She seemed to want a better sleep schedule, but it seemed like her work obligations create issues” (Age 47). The sixth barrier encompassed descriptions of home environment factors (e.g., light/noise/temperature/technology use) that made it difficult for the participant to sleep., “The participant listed various things that she believes impact her sleep, including light, noise, the weather (heat), the light from the TV set in the bedroom (what impacts her sleep more than anything else)” (Age 47). The seventh barrier included medical conditions/illnesses; for example, one interviewer described the following conversation: “… when asked if she ever had difficulty sleeping at night, she went on to tell us about a skin illness that she contracted which caused her not to be able to sleep/insomnia for about 1 year” (Age 44). Finally, the eight barrier related to women reporting the consumption of caffeine and certain foods. One interviewer mentioned “she likes to drink coffee and coke, but she cannot drink them in the evening because it will keep her awake” (Age 39).

Table 5 presents key subthemes related to sleep facilitators, including (1) medication/oral remedies/teas, (2) completing daily chores/preparing for the following day, (3) practicing relaxing activities before bed, (4) exercising/participating in activities during the day, (5) limiting foods/caffeine and (6) limiting noise or light exposure. We found that medication/oral remedies/tea was the top reported facilitator to achieving adequate or high-quality sleep. Examples of these included consuming homemade teas or medications obtained over the counter or prescribed by a clinician. As an interviewer recorded in fieldnotes, one woman described being prescribed medication but preferring home remedies instead, “Her doctor gave her drops for sleep, but she never took them, she prefers alternative medications like passionflower tea” (Age 60). Completing daily chores/preparing for the following day was the second commonly reported facilitator, such as one participant aged 49: “Before going to bed she likes to make sure her home is very clean, she leaves it this way so she can sleep, she won’t sleep if her house (especially the kitchen) isn’t clean at night.” The third most reported facilitator was practicing relaxing activities before bed. For instance, “She mentioned that another remedy she’s heard of is to take a shower in the night to help one relax” (Age 40). The remaining facilitators were much less frequently mentioned than the top 3. Facilitator 4 related to how exercising/participating in daily activities promoted sleep. For example, one interviewer highlighted the following: “a few tips she has heard to help people sleep include exercising and doing activities that make you very tired” (Age 47). The fifth most commonly reported facilitator was limiting certain foods and caffeine. For example, in conversation with a participant, an interviewer noted the following, “Avoid eating heavy foods at night” (Age 51). Another participant described the following to an interviewer: “Not drinking coffee, although coffee never affects her sleep” (Age 61). The final reported facilitator was limiting noise or light exposure at night. To illustrate this subtheme, one participant described the following to an interviewer: “Turning off the lights, getting rid of my distractions” (Age 51).

Table 6 describes women’s strategies to compensate for their lack of sleep. We identified three key subthemes: 1) consumption of caffeinated beverages, (2) napping and (3) resuming sleep after completing familial/household obligations. Consuming caffeinated drinks was the most commonly cited way to compensate for lack of sleep. The team’s fieldnotes described the following: “She told us that sometimes she has to buy a Monster or Redbull to help her stay awake.” (Age 39), and another fieldnote reported: “She also said that sometimes she has to drink a coke to wake herself up at work. (Age 60)” Although not as commonly reported by women, we also heard that women were napping to compensate for sleep. For example, from fieldnotes, one woman described how her work schedule allowed her to compensate for lack of sleep by taking naps at work: “By 2 PM she would go on a 2-hour lunch break and would take 10–15-minute naps to compensate for not sleeping enough the previous day” (Age 51). Finally, the resumption of sleep after completing early morning chores was another strategy employed by women. One interviewer captured an example of the following subtheme through fieldnotes “… she wakes up at 4 AM to prepare her husband’s breakfast/lunch for work, she goes back to bed at 5 AM and then wakes up again at 7 AM and brushes her teeth, etc., and helps her kids get ready for classes until 12 PM” (Age 40).

## Conclusion

In addressing the broader question of how psychosocial and cultural factors influence sleep and circadian health disparities (Sephton and Kay 2024), this study generated a few critical findings. First, our study highlighted the period of midlife as one where sleep disparities exist. Similar to other midlife populations (Arakane et al. 2011; Cuadros et al. 2012), many of the midlife women in the sample experienced poor sleep, including short sleep duration (approximately half of the sample did not obtain the recommended sleep) and insomnia-related sleep difficulties (40% of women self-reported experiencing a hard time falling asleep ≥ 3 days). Our results further demonstrated that these sleep challenges were not merely the result of individual choices or biological factors. Instead, sleep behaviors were strongly influenced by the women’s familial roles, especially caregiving responsibilities. Thus, as we think about addressing sleep disparities in midlife women, we need to more carefully consider their roles, both physical and emotional, within their families.

In many ways, the sleep challenges faced by working-class Mexican women are emblematic of sleep challenges faced by midlife women around the world. In our study population, women’s sleep was often constrained by early morning obligations, such as cooking for their families or preparing children for school, as well as work. In other cases, women stayed up late to wait for their adult children to return home from work or school. Further, we found that household and family stressors, including worries about family members’ well-being, were major contributors to poor sleep, particularly insomnia. These findings align with other studies of women in midlife (Roncoroni et al. 2022; Varma et al. 2020) and reinforce the idea that sleep is intricately tied to the family and social roles women play. Further, while socioeconomic status was not the central focus of our study, we observed significant disparities in sleep disturbances based on socioeconomic status. Women experiencing lower socioeconomic status reported safety concerns, such as fears about their homes collapsing or hearing gunshots at night, stressors not commonly reported by women of higher socioeconomic status. Again, these findings are not necessarily unique to the Mexican context and have been reported in other sleep disparity literature.

However, what is striking in this mixed-methods analysis is that while the ethnographic and epidemiologic assessment of sleep quality in this population came to very similar conclusions in some ways (i.e., a high proportion of women experiencing poor quality), the reasons for poor sleep quality looked a bit different. Specifically, the factors associated with sleep quality in the epidemiological analysis were more individual-based (particularly participants’ own chronic disease status and type of work), whereas almost all of the ethnographic findings were rooted in relationships with others. What this suggests is that neither method alone may be sufficient to fully understand the important drivers of sleep among midlife women and that the two types of data collection can feed into one another. For example, our findings suggest that future epidemiologic study visits should include more questions related to familial factors to uncover possible differences in sleep quality as well as other health outcomes. On the other side, findings from the epidemiological surveys suggest the need for more conversations about women’s own experiences with chronic disease, especially type 2 diabetes, in relation to sleep.

In contrast to our expectation, there was a relatively low prevalence of multi-generational households, with only 23% of our participants reporting living in such arrangements. Nonetheless, this is in line with recent estimates that one-fifth of Mexico’s population is now living in multi-generational households (Ideas Matter 2023). Of note, no specific themes or subthemes emerged from our data pointing to multi-generational households as a particular factor affecting sleep; essentially, all the women had familiar/caregiving responsibilities, whether they lived in these settings or not. Nonetheless, further study of the impacts of these housing arrangements is warranted before specific conclusions can be drawn.

Taken together our work suggests the need for sleep recommendations, including sleep hygiene principles, to be tailored specifically for midlife women, especially those with caregiving responsibilities. Notably, within this context of working-class midlife women, sleep hygiene recommendations to create a dark, quiet, separate space for sleep may not be feasible and, therefore, may not have much impact. Ideally, these recommendations should be centered on strategies that women are already employing. Indeed, women had a lot to say when it came to describing “sleep facilitators.” This included a lot of discussion on some form of “sleep remedy,” including medication (over the counter and prescribed) as well as a large variety of teas (e.g., passionflower, lettuce tea and more generally, “herbal tea”) (Zamora et al. 2024). In addition, strategies that women commonly employed to improve their nightly sleep were closely tied to family and household duties. For example, one of the most frequently cited facilitators was completing daily chores at home and mentally or physically preparing for the following day. Future research could explore how midlife women with caregiving responsibilities manage their sleep in the context of structural, social and familial demands and what supports might enhance their capacity to prioritize rest.

### Strengths and limitations

A key strength of this study was its mixed-methods design, which combined epidemiologic and ethnographic data. The ethnographic approach helped fill gaps that the epidemiological data alone could not capture, providing a more nuanced understanding of women’s sleep experiences. However, the study also had several limitations. Regarding the qualitative data, the subsample was purposively selected from the full study sample to ensure a balanced representation aross the entire age range. However, this subsampel differed in a few respects, such that the sample was slightly older on average. Our ability to conduct in-person ethnographic fieldwork was limited due to the COVID-19 pandemic, which prevented us from collecting additional sleep data and other relevant measures. Notably, we were unable to gather ethnographic sleep maps, which could have provided a more comprehensive understanding of how women in this group experience and navigate sleep in relation to their habits, daily routines and environmental factors. Another limitation was the modest quantitative sample size, which reduced the statistical power to detect differences. In addition, caution is needed when generalizing these results to other populations. Finally, a significant limitation was the way we assessed mental health status. We collected this information using close-ended questions about whether participants had a history of mental health conditions. This approach could have led to misclassification, underestimating the proportion of women with such a a history. Furthermore the epidemiological data were collected in 2019, prior to the onset of the COVID-19 pandemic. This timing which could have contributed to misclassification, as the pandemic significantly affected the mental health of adults. Despite these limitations, the results represent an important initial step toward developing a deeper understanding of sleep practices and experiences among middle-aged Mexican women. This knowledge can inform future research on sleep and intervention efforts for this demographic, future research on sleep and intervention efforts for this demographic understanding sleep patterns.

## Supplementary Material

2

1

**Supplementary material.** The supplementary material for this article can be found at https://doi.org/10.1017/slp.2025.5.

## Figures and Tables

**Figure 1.**
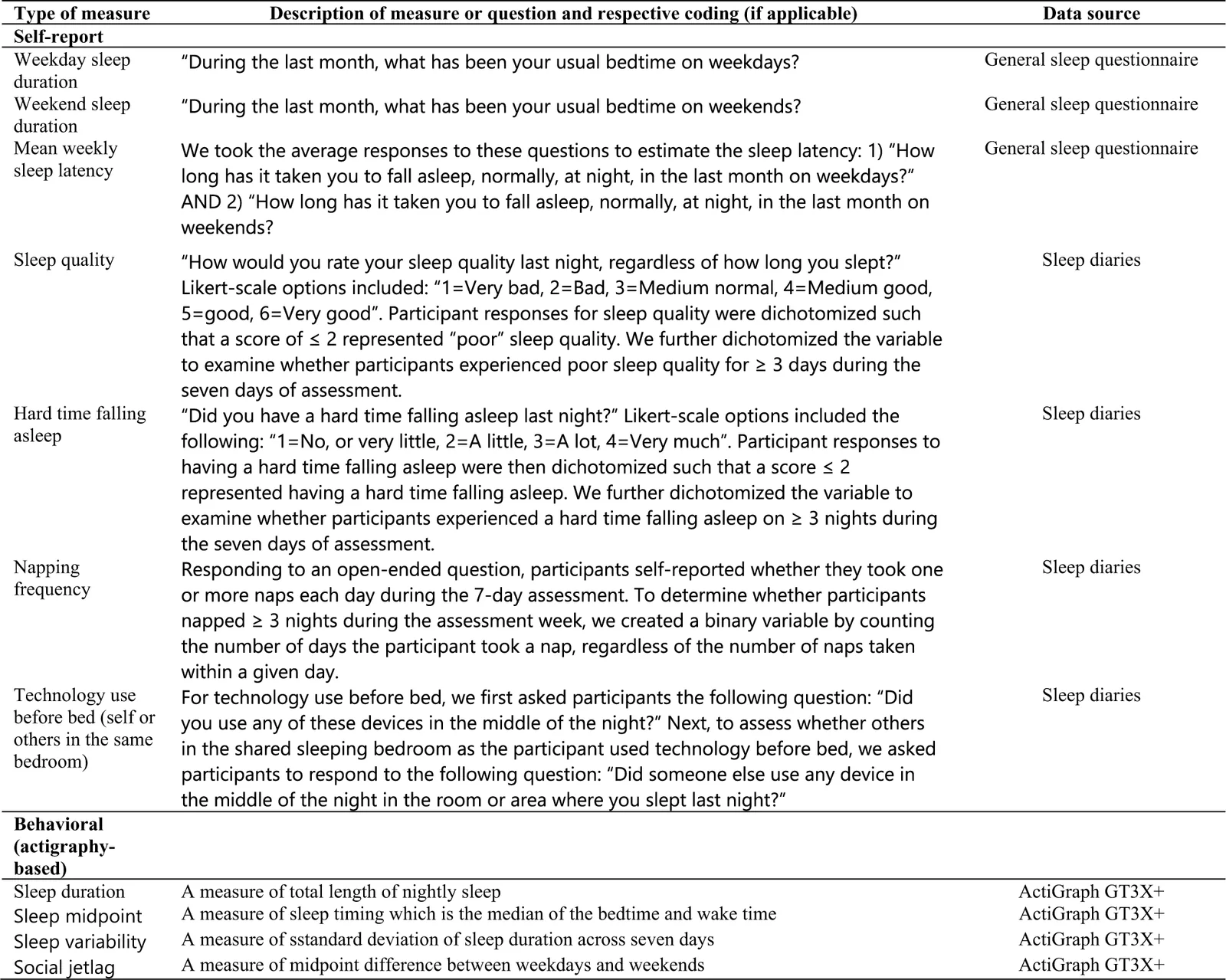
Description of sleep measures included in study.

**Table 1.** Demographic, clinical and sleep characteristics of the epidemiologic sample compared to the ethnographic sample

	Median (IQR) or Proportion	
	Epidemiologic sample (n = 120)	Ethnographic sample (n = 30)
**General demographics questionnaire data**		
Participant age	46.0 (8.0)	50.0 (9.0)
Type of job/occupation		
*Independent/self-owned*	33.6	26.7
*Company/organization*	33.6	26.7
*Dedicated to household, retired, or pensioned*	32.8	46.6
Number of bedrooms in household		
*1*	16.7	16.7
*2*	36.7	50.0
*3*	31.6	13.3
≥ *4*	15.0	20.0
Parity		
≤ *1*	14.2	10.0
*2*	36.8	50.0
≥ *3*	49.0	40.0
Household socioeconomic status		
*Lower*	50.8	50.0
*Middle/Higher*	49.2	50.0
Presence of household food insecurity		
Yes	43.2	52.0
*No*	56.8	48.0
Smoking behavior		
*Current smoker*	21.7	20.0
*Previous smoker*	14.2	6.6
*Never smoker*	30.0	36.7
*Unknown*	34.1	36.7
Presence of diabetes		
*Yes*	8.3	10.0
*No*	83.4	80.0
*Pre-diabetic*	8.3	10.0
Presence of mental health condition(s)		
*Yes*	12.6	10.0
*No*	87.4	90.0
Menopausal status		
*Peri*	23.3	33.3
*Pre*	16.7	30.0
*Post*	47.5	30.0
*Surgical*	12.5	6.7
**General sleep questionnaire data**		
Self-reported average sleep duration (hours per night)^c^	8.1 (1.8)	8.8 (1.5)
Time to fall asleep (min)^c^	10.0 (25.0)	15.0 (40.0)
**Sleep diary information (7 days)**		
Poor sleep quality ≥ 3 days per week^b^		
*Yes*	10.8	3.3
*No*	89.2	96.7
Hard time falling asleep ≥ 3 days per weekb		
*Yes*	43.3	43.3
*No*	56.7	56.7
Naps ≥ 3 days per weekb		
*Yes*	22.5	13.3
*No*	77.5	86.7
**Technology use before bed**		
*Participant uses technology before bed*	53.7	67.9
*Others use technology before bed*	45.2	51.7
**Sleep actigraphy data (7 days)**		
Sedentary time (min per day)	120.0 (180.0)	120.0 (180.0)
Sleep duration (hours per night)^a^	6.9 (1.4)	7.1 (1.7)
Sleep variability across the week (min)^a^	78.1 (48.6)	71.0 (47.7)
Sleep midpoint (decimal hour)^a^	3.4 (1.4)	3.9 (1.4)
Social jetlaga	0.9 (1.3)	0.6 (1.4)

a Data derived from actigraphy device (N = 120 for epidemiologic sample, N=30 for ethnographic sample).

b Data derived from sleep diaries (N = 120 for epidemiologic sample, N=30 for ethnographic sample).

c Data dervied from the general sleep questionnaire.

**Table 2.** Demographic and clinical correlates associated with self-reported (questionnaires and sleep diaries) and behavioral (actigraphy) sleep characteristics among the epidemiologic sample (N=120)

	N	Self-reported sleep duration (hr/night)	Time to fall asleep (min/ night)	Actigraphy-based sleep duration (hr/night)	Sleep variability across the week (min)	Sleep midpoint (decimal hr)	Social jetlag (hr)	Naps ≥ 3 days per week (%)	Poor sleep quality ≥ 3 days per week (%)	Hard time falling asleep ≥ 3 days per week (%)
		Median (Q1, Q3)	Median (Q1, Q3)	Median (Q1, Q3)	Median (Q1, Q3)	Median (Q1, Q3)	Median (Q1, Q3)			
Participant age-group, years										
*30–43*	46	8.2 (7.0, 9.0)	10.0 (5.0, 30.0)	6.6 (5.9, 7.4)	86.6 (58.9, 109.8)	3.3 (2.8, 4.1)	1.1 (0.7, 1.7)	26.3	2.6	47.4
*44–48*	36	8.0 (7.4, 8.8)	10.0 (5.0, 27.5)	7.0 (6.5, 7.5)	73.7 (47.3, 96.3)	3.4 (2.9, 4.0)	0.8 (0.1, 1.8)	22.7	11.4	40.9
*49–60*	38	8.6 (7.0, 9.0)	15.0 (5.0, 30.0)	7.0 (6.0, 7.8)	72.9 (54.1, 94.3)	3.5 (2.4, 4.1)	0.6 (0.2, 1.0)	18.4	18.4	42.1
*P*		0.41	0.75	0.20	0.08	0.90	**0.01**	0.70	0.08	0.82
Type of occupation/work										
*Independent/self-owned*	40	8.3 (7.4, 9.0)	15.0 (5.0, 30.0)	7.0 (6.4, 7.6)	75.3 (55.3, 94.8)	3.6 (2.4, 4.0)	0.6 (0.0, 1.4)	17.5	5.0	42.5
*Company/organization*	40	7.5 (6.5, 8.7)	10.0 (2.0, 20.0)	6.5 (5.8, 7.3)	82.7 (48.1, 110.0)	3.1 (2.6, 4.1)	1.0 (0.4, 1.7)	20.0	7.5	42.5
*Dedicated to household, retired, or pensioned*	39	8.5 (7.8, 9.5)	15.0 (5.0, 30.0)	6.9 (6.3, 7.8)	81.5 (58.9, 95.0)	3.5 (3.0, 4.4)	0.9 (0.2, 1.7)	30.8	20.5	43.6
*P*		**0.0044**	**0.0118**	0.11	0.77	0.52	0.33	0.32	0.06	0.99
Number of bedrooms in household										
*1*	20	8.4 (7.4, 8.9)	10.0 (5.0, 30.0)	6.2 (5.7, 7.4)	83.0 (55.8, 104.2)	3.7 (3.0, 4.4)	1.0 (0.5, 1.7)	20.0	15.0	50.0
*2*	44	8.5 (7.7, 9.0)	10.0 (5.0, 30.0)	7.2 (6.5, 7.9)	80.5 (55.7, 101.9)	3.5 (2.7, 4.1)	1.0 (0.1, 1.5)	27.3	11.4	52.3
*3*	38	7.8 (7.0, 9.3)	15.0 (5.0, 30.0)	6.9 (6.4, 7.7)	72.5 (49.7, 95.0)	3.4 (2.9, 4.0)	0.9 (0.3, 1.4)	15.8	7.9	42.1
≥ *4*	18	8.0 (7.0, 8.8)	10.0 (5.0, 25.0)	6.5 (6.2, 7.0)	78.9 (62.8, 93.7)	2.8 (2.3, 3.7)	0.6 (0.1, 2.0)	27.8	11.1	16.7
*P*		0.53	0.74	0.05	0.87	0.76	0.41	0.59	0.87	0.07
Parity										
*1*	17	7.8 (7.5, 8.5)	5.0 (3.0, 15.0)	7.2 (6.1, 7.8)	83.8 (68.5, 107.8)	3.5 (3.1, 4.1)	0.9 (0.1, 2.2)	23.5	11.8	41.2
*2*	44	8.4 (7.5, 9.0)	10.0 (5.0, 25.0)	6.9 (6.3, 7.4)	75.8 (60.5, 102.8)	3.1 (2.5, 4.0)	0.8 (0.2, 1.5)	29.6	11.4	38.6
≥ *3*	59	8.0 (7.0, 9.0)	15.0 (5.0, 30.0)	6.8 (6.0, 7.6)	78.2 (47.3, 98.4)	3.6 (2.9, 4.1)	0.9 (0.3, 1.6)	17.0	10.2	47.5
P		0.31	0.09	0.82	0.63	0.52	0.78	0.31	0.97	0.65
Household socioeconomic status										
*Lower*	61	8.3 (7.0, 9.0)	10.0 (5.0, 30.0)	7.1 (6.3, 7.7)	82.1 (62.0, 98.4)	3.6 (2.7, 4.1)	0.8 (0.2, 1.5)	23.0	14.8	39.3
*Middle/Higher*	59	8.0 (7.3, 9.1)	10.0 (5.0, 30.0)	6.7 (6.1, 7.4)	73.3 (46.8, 103.5)	3.2 (2.8, 4.0)	0.9 (0.3, 1.7)	22.0	6.8	47.5
*P*		0.58	0.48	0.10	0.20	0.20	0.52	0.90	0.16	0.36
Household food insecurity										
*Yes*	48	8.1 (7.0, 9.0)	15.0 (5.0, 30.0)	6.8 (6.1, 7.4)	86.3 (56.9, 107.8)	3.5 (2.4, 4.1)	1.1 (0.4, 1.7)	20.8	12.5	54.2
*No*	63	8.0 (7.1, 9.0)	10.0 (5.0, 25.0)	6.9 (6.1 7.6)	73.1 (47.6, 98.4)	3.2 (2.8, 4.0)	0.9 (0.2, 1.4)	23.8	7.9	33.3
*P*		0.74	0.07	0.39	0.10	0.64	0.33	0.71	0.42	**0.02**
Smoking behavior										
*Current smoker*	26	8.0 (7.0, 8.8)	10.0 (5.0, 30.0)	6.9 (6.2, 7.3)	73.5 (58.4, 107.4)	3.4 (2.9, 4.6)	1.3 (0.5, 1.7)	26.9	19.2	65.4
*Previous smoker*	17	8.0 (7.3, 8.9)	15.0 90 (5.0, 30.0)	6.8 (6.0, 7.6)	83.8 (57.3, 94.6)	3.4 (2.6, 4.1)	0.6 (0.2, 1.5)	29.4	5.9	29.4
*Never smoker*	36	8.0 (7.3, 9.0)	12.5 (5.0, 37.5)	6.7 (6.1, 7.8)	85.0 (65.8, 111.5)	3.7 (2.8, 4.1)	0.8 (0.1, 1.5)	19.4	8.3	41.7
*Unknown*	41	8.3 (7.1, 9.0)	10.0 (5.0, 30.0)	7.1 (6.4, 7.5)	73.3 (44.5, 95.0)	3.2 (2.4, 3.7)	0.9 (0.2, 1.1)	19.5	9.8	36.6
*P*		0.94	0.96	0.94	0.43	0.40	0.30	0.76	0.45	0.06
Presence of diabetes										
*Yes*	10	7.9 (7.3, 8.8)	10.0 (5.0, 30.0)	7.0 (6.2, 7.6)	83.8 (62.0, 97.6)	3.1 (2.7, 3.6)	0.7 (0.1, 1.7)	30.0	20.0	50.0
*No*	100	8.2 (7.0, 9.0)	10.0 (5.0, 30.0)	6.6 (5.7, 7.2)	79.6 (51.2, 101.9)	3.6 (2.8, 4.1)	0.9 (0.3, 1.6)	23.0	9.0	43.0
*Pre-diabetic*	10	8.4 (8.0, 9.0)	10.0 (5.0, 20.0)	6.6 (6.2, 7.5)	70.1 (60.6, 85.8)	2.7 (2.4, 3.1)	0.3 (0.1, 0.6)	10.0	20.0	40.0
*P*		0.80	0.85	0.44	0.36	**0.04**	0.05	0.53	0.35	0.89
Presence of mental health condition(s)										
*Yes*	15	8.0 (7.3, 9.5)	30.0 (2.0, 60.0)	6.9 (6.2, 7.6)	102.1 (83.8, 115.7)	3.6 (2.4, 4.3)	0.6 (0.1, 1.6)	26.7	26.7	40.4
*No*	104	8.1 (7.0, 9.0)	10.0 (5.0, 30.0)	7.0 (6.0, 7.7)	73.2 (48.1, 95.1)	3.4 (2.8, 4.1)	0.9 (0.3, 1.5)	21.2	8.7	60.0
*P*		0.81	0.48	0.75	**0.0022**	0.38	0.78	0.62	**0.03**	0.15
Menopausal status										
*Peri*	28	8.6 (7.3, 9.3)	10.0 (3.5, 30.0)	7.1 (6.3, 7.6)	84.8 (53.7, 103.5)	4.0 (3.0, 4.6)	0.9 (0.0, 1.5)	25.0	14.3	39.3
*Pre*	57	8.0 (7.3, 8.8)	10.0 (5.0, 30.0)	7.2 (5.9, 7.8)	82.1 (62.4, 107.4)	3.4 (2.8, 4.0)	1.1 (0.5, 1.9)	28.1	7.0	42.1
*Post*	20	8.4 (6.8, 9.1)	10.0 (4.0, 30.0)	6.8 (6.2, 7.4)	66.8 (49.0, 85.3)	3.3 (3.5, 4.0)	0.6 (0.3, 1.1)	15.0	15.0	40.0
*Surgical*	15	8.0 (7.5, 9.0)	10.0 (5.0, 30.0)	6.8 (6.0, 7.4)	73.3 (47.6, 93.7)	2.8 (2.4, 3.6)	0.8 (0.2, 1.0)	6.7	13.3	60.0
*P*		0.61	0.98	0.49	0.28	0.07	0.16	0.27	0.64	0.57

Bold P-values denote P < 0.05; Note: Sample sizes vary across groups, with some groups having fewer than 120 observations; Abbreviations: Q1: quartile 1: Q3: quartile 3.

**Table 3.** Why is sleep needed?

Subthemes	Representative quote from researcher fieldnotes
*Energy and well-being*	The participant said that sleep is needed to move forward, feel good, healthy. (Age 39)
	To the participant, sleep is incredibly important. Without sleep we lack energy, we can’t function and do our regular activities, we feel tired all of the time. (Age 51)
	Sleep is essential to the participant; she wakes up feeling energized when she sleeps well ... (Age 60)
	She mentioned that sleep is important to help the body feel relaxed, to have energy for the next day ... she also said feeling tired and lacking energy (Age 40)
	It is sleeping that helps you recover so that the body has energy, she told us that it is something that the body asks for ... she considers that sleep should be sacred like the food we eat (Age 49)
*To avoid poor mental/ physical health*	She mentioned that if someone lacks sleep, they can experience a heart attack, their vision and ability to hear can become impaired. She told us she experienced tachycardia from her lack of sleep and thinks sleep is critical for the human race. (Age 39)
	Whenever she lacks sleep, she feels dizzy the next day; she feels like her blood pressure increases, she gets moody. (Age 54)
	Back when she worked, if she lacked sleep, she would wake up with red eyes, and it would be very hard to focus. When she lacks sleep and is at home, she tends to get headaches, her eyes hurt, and she feels upset/angry all of the time. (Age 52)
	This participant, like others, mentioned that when others lack sleep it might lead to illnesses and issues with anxiety/nerves and not being able to sleep. (Age 52)
	When she doesn’t sleep enough, see gets angry, upset, because she feels like the world is responsible for her not being able to sleep enough. (Age 40)
*To perform at work*	One thing she said that stuck with me when I asked what happens to folks that don’t sleep enough was that they do not perform. (Age 50)
	One thing I found interesting was that she told me that sometimes she will fall asleep at work if she didn’t sleep well the night before. (Age 47)
	When she has not slept enough, her work is greatly affected ... (Age 58)
	The participant said that she is not able to function 100% if she does not sleep enough and goes to work the next day. She told us that sometimes she has to buy a Monster or Redbull to help her stay awake. During her first two months of work/ being a widow, she started falling asleep at work due to not sleeping enough the night before, so she could not function properly. (Age 39)
	The last thing she said about sleep was that sleep is essential; if you do not sleep enough, you can’t shine at work and people need to sleep to be able to function the next day. (Age 59)
*To avoid accidents*	She also said that she thinks lacking sleep will lead to headaches/migraines/accidents at home or out in the street (Age 47)
	She linked the importance of sleeping with having energy to do things and repeated something we have previously heard from participants, which is that if you don’t sleep enough you can get into accidents (Age 43)
	When I asked her how her work used to be affected if she lacked sleep, she said that it felt so heavy, once she burned her hand for being so tired at work and not being able to focus. (Age 56)

**Table 4.** Women’s perceptions of sleep barriers faced by self or others

Subthemes*	Representative quote from researcher fieldnotes
*Family*	“Worrying about her children and their obligations, such as schoolwork that stresses them out or keeps them up at night.” (Age 47)
	“If she has to take care of her dad (i.e., doctor appts), she wakes up at 5–630 AM ... when she has to be up early, she has issues sleeping the night before and gets up about 3 times per night.” (Age 51)
	“She wakes up at 4 AM to prepare her husband’s breakfast/lunch for work, she goes back to bed at 5 AM and then wakes up again at 7 AM and brushes her teeth, etc., and helps her kids get ready for classes until 12 PM. If her husband doesn’t have to work, she wakes up later on those days” (Age 40)
	“Her sleep schedule is impacted by when her kids go to school, she told us that she wakes up early to send her child to school in the mornings. This was very clear, because she told us that the days her child does not go to school, she wakes up at a different time.” (Age 45)
	“Worrying about a family member coming home late from work ... which is true for her, since her son gets home late from work ... she told us that her daughter gets home super late at night (1:30 AM) and she won’t sleep until her daughter gets home.” (Age 49)
*Economic situation*	“She noted that she has difficulty sleeping whenever she has financial problems or is stressed from obligations.” (Age 60)
	“She cannot take naps during the day because of her current (financial) situation” (Age 60)
	“Worrying that the house she lives in will come falling on them or her son not sleeping well at night” (Age 61)
	“Some of the factors that impact her sleep include financial concerns, those that affected her immediate household during the pandemic” (Age 51)
	She told me that she thinks her issues with sleep are driven by economic problems at home” (Age 43)
*Stress/rumination*	“Things that impact her sleep include stress, worrying about things ... things happening in the world” (Age 40)
	“There have been times when she has anxiety at night, and she tosses, and turns, but this happens rarely.” (Age 50)
	“The things that affect her sleep are being sad about the pandemic, feeling anguish over people dying, worrying about her loved ones, and not being able to leave the house to do anything.” (Age 56)
	“She told us that stress and thinking about things can keep her up until midnight on some nights” (Age 43)
	“She repeated to us that she would not sleep because she was really worried about getting sick, and stressed” (Age 45)
*Unexplained Insomnia*	“The participant told us that she used to sleep well at night but has experienced insomnia for the past five years and has difficulty sleeping.” (Age 54)
	“From about age 45 through the present day, she hasn’t been sleeping well, and she thinks she has insomnia since she only gets like 30 minutes of sleep (Age 52)
	“She has always had issues sleeping throughout her life. She told us that she has suffered a lot in the past from insomnia.” (Age 41)
	“She also told me that between Dec-Feb 2021 she has a lot of issues sleeping, she mentioned in her own words that she was experiencing insomnia” (Age 43)
	“Though she hadn’t mentioned her dad before, she mentioned that her dad suffered a lot from insomnia and that she thinks she got her sleep habits from him.” (60)
*Job/occupation schedule or stress*	“Later she mentioned that she thinks that the pandemic isn’t the reason why her sleep has changed, she lost her job and that affected her sleep the most.” (Age 47)
	“Things that impact her sleep include ... work/jobs changing dramatically” (Age 40)
	“She seemed to want a better sleep schedule, but it seemed like her work obligations create issues.” (Age 47)
	“Sometimes she naps during the day, but only when she is not stressed about work.” (Age 52)
*Home environment factors (i.e., light/noise/ temperature/technology use)*	“Things that affect the participant are noises in the neighborhood – music, dogs, car alarms ... ” (Age 53)
	“Usually, noises or light being reflected from a cell phone (affects the participant’s ability to sleep)” (Age 39)
	“She mentioned that factors that negatively affect her ability to sleep include her husband snoring ... as well as the noise from the airplanes that fly over her home.” (Age 54)
	“The participant listed various things that she believes impact her sleep, including light, noise, the weather (heat), the light from the TV set in the bedroom (what impacts her sleep more than anything else).” (Age 47)
	“I asked her what type of factors impact her sleep and that of others, she mentioned noises outside for her and for others noises, but gave examples like gunshots, yelling, ambulance, etc.” (Age 47)
*Medical conditions/ illnesses*	“She also mentioned that if she has diarrhea or has stomach aches, she can’t sleep well” (Age 53)
	“She said that problems ... illness ... keep her from sleeping at night.” (Age 49)
	“ ... when asked if she ever had difficulty sleeping at night, she went on to tell us about a skin illness that she contracted which caused her not to be able to sleep/insomnia for about 1 year.” (Age 44)
	“ ... she had a lot of thoughts to offer regarding what impacts the sleep of other people, such as pains” (Age 52)
	“One of her younger sisters also had COVID and suffered a lot to the point of being hospitalized and was even put on a ventilator. Her sister had bad anxiety, she felt like she was going to die, and that impacted her sleep a lot.” (Age 50)
*Caffeine/Food*	“She told me that she is trying to cut back on the coffee because it affects her falling asleep.” (Age 58)
	“She said she likes to drink coffee and coke, but she cannot drink them in the evening because it will keep her awake.” (Age 39)
	“ ... she mentioned that she wakes up about three times per week; she wakes up about 2–3 times per night. She thinks the coffee is what makes her get up each time.” (Age 59)
	“She mentioned that her younger son’s sleep is bothered by candy, or overeating food at night ... ” (Age 47)
	“Things that might impact the sleep of others include ... chocolate, and heavy meals (i.e., barbacoa, carnitas, tacos, etc.) making it difficult to sleep due to lack of proper digestion/feeling very full.” (Age 39)

* Subthemes not mutually exclusive and may appear under multiple codes.

**Table 5.** Women’s perception of sleep facilitators that they have tried or heard about from others

Subthemes	Representative quote from researcher fieldnotes
*Medication/oral remedies/ teas*	“Her doctor gave her drops for sleep, but she never took them, she prefers alternative medications like passionflower tea” (Age 60)
	“Tips she has heard can help people fall asleep include ... lettuce tea, a cup of milk” (Age 50)
	“Remedies that help with sleep issues include blossom tea, putting lettuce under a pillow and taking medications.” (Age 54)
	“She mentioned a few medications ... Azcetazolamide which she took when she had her son, and Hydroxyzine, which her doctor prescribed her” (Age 56)
	“She shared the name of a medication that is used for relaxing and sleeping, but it was hard to understand the name. She told us that her sister works in a pharmacy, and she recommended it to her, so she asked her doctor, and he also said it was good ... She also said that valerian is good for sleeping” (Age 45)
*Completing daily chores/ preparing for the following day*	“Before going to bed she likes to make sure her home is very clean, she leaves it this way so she can sleep, she won’t sleep if her house (especially kitchen) isn’t clean at night” (Age 49)
	“She mentioned needing to do some activities in order to help her be able to sleep ... ” (Age 58)
	“Before going to bed each night ... lays in bed and thinks about what she is going to make for dinner the following day, usually just thinking about what her day will look like helps her fall asleep” (Age 59)
	“She has heard that doing activities makes others tired, the example she gave us was ironing clothes ... in general, doing things that make us tired ... ” (Age 58)
*Practicing relaxing activities before bed*	“Things she knows can help people fall asleep are ... listening to music, this one works for her” (Age 39)
	“If she has a hard time falling asleep, she reads a book” (Age 53)
	“She told us that some remedies that help with sleep are ... the chiropractor which would help her relax, homeopathy, listening to the gospel of the lord, seeing specialists that can help with sleep problems ... ” (Age 52)
	“She mentioned that another remedy she’s heard of is to take a shower in the night to help one relax” (Age 40)
	“The third activity that she mentioned to us is that she watches TV, which is common. For the first time I asked what she watches, to which she replied that she watches a soap opera, which I consider to be a very common activity, watching TV before going to sleep.”(Age 47)
*Exercising/participating in activities during the day*	“She also mentioned a few tips she has heard to help people sleep, including exercise and doing activities that make you very tired (Age 47)
	“She told us that one of her colleagues told her she was practicing yoga, that it was relaxing ... ” (Age 40)
	“Tips she has heard can help people fall asleep include doing exercises that help relax you” (Age 50)
	“Things she has heard can help with sleep are exercising ... and yoga” (Age 51)
	“When asked what advice she has heard to help people sleep, she mentioned ... being physically active ... ” (Age 44)
*Limiting foods/caffeine*	“Avoid eating heavy foods at night” (Age 51)
	“Not drinking coffee, although coffee never affects her sleep” (Age 61)
*Limited noise or light exposure*	“Turning off the lights, getting rid of any distractions” (Age 51)

**Table 6.** Strategies women practice to compensate for lack of sleep

Subthemes	Representative quote from researcher fieldnotes
Consumption of caffeinated beverages	“She told us that sometimes she has to buy a Monster or Redbull to help her stay awake. During her first two months of work/being a widow, she started falling asleep at work due to not sleeping enough the night before, so she could not function properly.” (Age 39)
	“When I asked what happens to her at work if she lacks sleep, she said she does the work tired, and without energy. She also said that sometimes she has to drink a coke to wake herself up at work.” (Age 60)
Napping	“When I asked her how her previous work would be affected if she lacked sleep, she said not much because her job was really relaxing (computer job), she would get to work, drink coffee/tea and 10 AM she would have breakfast and then sit and revise documents on the computer. By 2 PM she would go on a 2 hour lunch break and would take 10–15 minute naps to compensate for not sleeping enough the previous day.” (Age 51)
Resuming sleep after completing familial/ household obligations	“ ... she wakes up at 4 AM to prepare her husband’s breakfast/lunch for work, she goes back to bed at 5 AM and then wakes up again at 7 AM and brushes her teeth, etc., and helps her kids get ready for classes until 12 PM” (Age 40)
	“Her sleep schedule is impacted by when her kids go to school, she told us that she wakes up early to send her child to school in the mornings. This was very clear, because she told us that the days her child does not go to school, she wakes up at a different time.” (Age 45)

## Data Availability

The data that support the findings of this study are available through a secure server at the University of Michigan, but subject to IRB approval and a data use agreement.
