# Supplementary material for: Social drivers of sleep experiences: Conversations with midlife working-class women from Mexico city
Source: Res Dir Sleep Psychol. Author manuscript; Available in PMC 2026 Nov 7. (PMC13742513; doi:10.1017/slp.2025.5)
Supplement: Supplementary file 2 [file NIHMS2215893-supplement-2.docx]

**Supplemental Table 1.** Study codebook with codes, definitions, and interview topics connected to codes

| **Code** | **Definition** |
| --- | --- |
| Energy and well-being | Descriptions of sleep being necessary to energize, rejuvenate the body, as well as descriptions related to how a previous night of sleep can allow one to feel rested the following day |
| To avoid poor mental/physical health | Descriptions of sleep being necessary to avoid poor mental or physical health outcomes |
| To perform at work | Descriptions of sleep being necessary to perform well at work and to avoid accidents/mistakes at work. |
| To avoid accidents | Descriptions of sleep being necessary to avoid accidents within and outside of the home, as well as at work |
| Family | Descriptions or narratives about barriers to sleep related to the family including spending time with family, family duties or obligations, thinking about family, etc. |
| Economic situation | Descriptions or narratives indicating the participant's economic situation is a barrier to sleep, this can include worries about their economic situation or more infrastructural economic barriers to sleep |
| Stress/rumination | Descriptions indicating that the participant was thinking or overthinking something that created a barrier to sleep, including thoughts or activities about the next day |
| Unexplained insomnia | Descriptions indicate the participant "just can't sleep" but does not know why. In addition, any descriptions where participants explicitly mention insomnia, or symptoms of insomnia, including distress over having a hard time falling or staying asleep. |
| Home environment factors | Quotes indicating that home environment factors such as light, noise, temperature, or technology use (i.e., phones, tablets, TV, computer, etc.) is a barrier to sleep |
| Job/occupation schedule or stress | Descriptions indicating that the job/occupation schedule or stress of the focal participant or family members is a barrier to sleep |
| Medical conditions/illnesses | Descriptions or narratives about how medical conditions or illnesses (including COVID) create issues with sleeping |
| Caffeine/food | Descriptions or narratives indicating that caffeine or consumption of foods is a barrier to sleep. Can include coffee, caffeinated soda, caffeinated tea, and other types of caffeine, as well as any mentions of food items that create a sleep barrier |
| Medication/oral remedies/teas | Descriptions of using medication or home remedies that are taken orally (including tea) to facilitate sleep |
| Completing daily chores/preparing for the following day | Descriptions or narratives about the ability to sleep as soon as the participant has completed daily household chores or feels prepared for the next day |
| Practicing relaxing activities | Descriptions or narratives about nightly activities done before bed help the participant to relax and settle in for sleep, such as reading a book, taking a shower, watching a show, etc. |
| Exercising and participating in activities during the day | Descriptions or narratives related to participants needing to exercise or participate in activities during the day in order to feel tired and able to sleep at night |

**Supplemental Table 2.** Participant profiles obtained from ethnographic interview

| **Participant interview #** | **Age** | **Job/occupation industry** | **Description of job/occupation (if applicable)** | **# People living in the household** | **Household composition**^1^ | **# People sharing a bedroom with the participant** |
| --- | --- | --- | --- | --- | --- | --- |
| 1 | 44 | Dedicated to household | 5 years dedicated to the household | 4 | Single-family | 2 |
| 2 | 50 | Dedicated to household | Dedicated to household, but also a caretaker as needed | 2 | Single-family | 0 |
| 3 | 54 | Small business owner | Owns a beauty salon | 7 | Multi-family | 1 |
| 4 | 40 | Blue collar services | General helper | 4 | Single-family | 1 |
| 5 | 47 | Blue collar services | House keeper | 3 | Single-family | 1 |
| 6 | 41 | Clerical/office work | Medical assistant in a hospital | 10 | Multi-family | 1 |
| 7 | 43 | Clerical/office work | Secretary at a government office | 6 | Multi-family | 3 |
| 8 | 52 | Small business owner | Owns a sewing/tailoring shop | 4 | Single-family | 1 |
| 9 | 45 | Dedicated to household | 26 years dedicated to household | 6 | Multi-family | 1 |
| 10 | 47 | Blue collar services | House keeper | 4 | Single-family | 1 |
| 11 | 61 | Dedicated to household | Pensioned | 3 | Single-family | 2 |
| 12 | 60 | Blue collar services | House keeper | 3 | Single-family | 2 |
| 13 | 40 | Clerical/office work | Receptionist | 7 | Single-family | 1 |
| 14 | 43 | Dedicated to household | Dedicated to the household for an unknown period | 5 | Single-family | 1 |
| 15 | 49 | Clerical/office work | Works in the formal sector | 5 | Single-family | 1 |
| 16 | 47 | Clerical/office work | Secretary in an office | 4 | Single-family | 1 |
| 17 | 52 | Dedicated to household | Dedicate to household, but also a vendor of home goods | 3 | Single-family | 2 |
| 18 | 58 | Clerical/office work | Business office worker | 4 | Single-family | 1 |
| 19 | 39 | Blue collar services | Security guard at a local shop | 3 | Single-family | 2 |
| 20 | 50 | Clerical/office work | Clerical work at the Social Security office | 5 | Single-family | 1 |
| 21 | 54 | Dedicated to household | 20 years dedicated to household | 4 | Single-family | 1 |
| 22 | 59 | Blue collar services | Cashier at a grocery store | 3 | Single-family | 1 |
| 23 | 53 | Blue collar services | Housekeeper | 3 | Single-family | 1 |
| 24 | 56 | Dedicated to household | 24 years dedicated to household | 3 | Single-family | 1 |
| 25 | 54 | Dedicated to household | 19 years dedicated to household | 9 | Multi-family | 1 |
| 26 | 51 | Clerical/office work | Social worker | 4 | Single-family | 1 |
| 27 | 56 | Dedicated to household | 2 years dedicated to household | 6 | Single-family | 1 |
| 28 | 52 | Clerical/office work | Administrative assistant | 4 | Single-family | 1 |
| 29 | 51 | Clerical/office work | Clerical worker in an accounting office | 11 | Multi-family | 3 |
| 30 | 52 | Dedicated to household | 12 years dedicated to household | 10 | Multi-family | 1 |

^1^Multi-family households include either multi-generational household (e.g., participant living with offspring and their grand offspring OR participant living with their parents, etc.) or participant living with multiple families within the same generation (e.g., the participants sisters’ family, etc.)
